# Therapeutic effects of the mitochondrial ROS-redox modulator KH176 in a mammalian model of Leigh Disease

**DOI:** 10.1038/s41598-017-09417-5

**Published:** 2017-09-15

**Authors:** Ria de Haas, Devashish Das, Alejandro Garanto, Herma G. Renkema, Rick Greupink, Petra van den Broek, Jeanne Pertijs, Rob W. J. Collin, Peter Willems, Julien Beyrath, Arend Heerschap, Frans G. Russel, Jan A. Smeitink

**Affiliations:** 1 Department of Pediatrics, Radboud Center for Mitochondrial Medicine, Radboud University Medical Center, Nijmegen, The Netherlands; 2 Department of Pharmacology and Toxicology, Radboud Center for Mitochondrial Medicine, Radboud University Medical Center, Nijmegen, The Netherlands; 3 Department of Radiology and Nuclear Medicine, Radboud Center for Mitochondrial Medicine, Radboud University Medical Center, Nijmegen, The Netherlands; 4 Department of Human Genetics, Radboud Institute for Molecular Life Sciences, Radboud University Medical Center, Nijmegen, The Netherlands; 5 Department of Biochemistry, Radboud Center for Mitochondrial Medicine, Radboud University Medical Center, Nijmegen, The Netherlands; 6 Khondrion BV, Nijmegen, The Netherlands

## Abstract

Leigh Disease is a progressive neurometabolic disorder for which a clinical effective treatment is currently still lacking. Here, we report on the therapeutic efficacy of KH176, a new chemical entity derivative of Trolox, in *Ndufs4* ^−/−^ mice, a mammalian model for Leigh Disease. Using *in vivo* brain diffusion tensor imaging, we show a loss of brain microstructural coherence in *Ndufs4* ^−/−^ mice in the cerebral cortex, external capsule and cerebral peduncle. These findings are in line with the white matter diffusivity changes described in mitochondrial disease patients. Long-term KH176 treatment retained brain microstructural coherence in the external capsule in *Ndufs4* ^−/−^ mice and normalized the increased lipid peroxidation in this area and the cerebral cortex. Furthermore, KH176 treatment was able to significantly improve rotarod and gait performance and reduced the degeneration of retinal ganglion cells in *Ndufs4* ^−/−^ mice. These *in vivo* findings show that further development of KH176 as a potential treatment for mitochondrial disorders is worthwhile to pursue. Clinical trial studies to explore the potency, safety and efficacy of KH176 are ongoing.

## Introduction

Mitochondrial diseases are heterogeneous multisystem disorders with a prevalence of 1 in 5,000 live births^1, 2^. Frequently these inborn errors of energy metabolism are caused by nuclear DNA (nDNA) or mitochondrial DNA (mtDNA) mutations affecting the phosphorylation (OXPHOS) system^3–6^. Complex I or NADH:ubiquinone oxidoreductase (EC 1.6.5.3) is the first and largest OXPHOS enzyme complex consisting of 37 nDNA and 7 mtDNA encoded protein subunits^7–9^. It is the most common origin for mitochondrial abnormalities^10, 11^ and both nDNA and mtDNA mutations can give rise to increased reactive oxygen species (ROS) production, altered redox status, and abnormal mitochondrial architecture^12–14^.

Leigh Disease (LD) is one of the many phenotypic appearances with which OXPHOS diseases can present^15^. The incidence of LD is approximately 1 in 77,000 live births and symptoms typically begin early in life^16, 17^. Neuro-imaging in LD patients reveal bilateral lesions in the basal ganglia, thalami, brainstem and dentate nuclei^18^. The prognosis for individuals with LD is generally poor and most patients decease at an early age^19, 20^. There is an unmet medical need to develop clinical effective treatments for LD and other mitochondrial disorders. Several potential new treatment strategies are under pre-clinical development^21–24^. Promising results of pharmacological or genetic interventions in mice have been described, acting on mitochondrial biogenesis, mitochondrial morphology, rapamycin mTOR signaling or oxidative stress^25–33^. We observed that oxidation of the ROS sensors hydroethidine and 5- (and -6)-chloromethyl-2′,7′-dichlorodihydrofluorescein diacetate (CM-H2DCFDA) was significantly increased in genetically characterized primary skin fibroblasts derived from LD patients with isolated complex I deficiency^34, 35^. The α-tocopherol derivative Trolox normalized these increased levels of CM-H2DCF oxidation in patient’s fibroblasts^36^. Phenotypic effects of Trolox and four newly developed Trolox variants (KH001–KH004) in LD patient cells were evaluated by using a machine learning strategy^37^. One of the Trolox variants (KH003) effectively scavenged ROS and increased the maximal activity of complex I, complex IV, and citrate synthase, suggesting that this variant might be a promising candidate for the development of therapeutic strategies in mitochondrial disorders. Encouraged by these findings a lead-optimization program was started to improve the drug-likeness properties of the candidate^37, 38^. Since ROS increase and redox signaling perturbation are common consequences of OXPHOS deficiencies, the 226 new chemical entities generated were evaluated in parallel for their potency and efficacy in scavenging cellular ROS and protecting patient-derived cells against Redox perturbation^13, 39–42^. Ultimately, KH176 was selected as the lead compound for further studies, based on its potency, efficacy in ROS-redox assays but also importantly based on its physicochemical properties such as oral bioavailability, cell and blood-brain barrier permeability, water solubility and chemical stability^43^. The effect of the compounds on OXPHOS enzymes activity was not included in the lead optimization program. It is worth mentioning that while KH176 is more potent when compared to the hit compound KH003 in the ROS and redox assay, it didn’t increase the activity of complex I activity *in vitro* (unpublished data) as opposed to KH003^38^.

This study examines the therapeutic effect of KH176 in a mammalian model of LD, the NADH dehydrogenase [ubiquinone] iron-sulfur protein 4 (*Ndufs4*) knockout (*Ndufs4* ^−/−^) mouse model^44^. Additionally, for the first time, brain microstructural coherence has been evaluated in this mouse model. Primary muscle and skin fibroblasts from *Ndufs4* ^−/−^ mice display increased ROS levels and aberrant mitochondrial morphology^45^. Furthermore, immortalized *Ndufs4* ^−/−^ mouse embryonic fibroblasts showed reduced oxygen consumption, elevated NAD^+^/NADH ratio and lactate production^46^. These findings indicate a role of increased ROS formation and altered redox balance in the disease pathogenesis in this animal model. *Ndufs4* ^−/−^ mice show a progressive neurodegenerative phenotype characterized by marked bilateral lesions in the vestibular nuclei, lethargy, ataxia, weight loss, epilepsy and ultimately death at a median age of postnatal day (PD) 45^44, 47, 48^. In *Ndufs4* ^−/−^ mice, protein succination as a result of Krebs cycle inhibition is increased in the brainstem, specifically in the vestibular nuclei^49^. This is the most affected region and it has been shown that *Ndufs4* ^−/−^ mice typically die of respiratory failure attributed to vestibular nucleus pathology^50^. Recently, increased oxidative stress has been determined in *Ndufs4* ^−/−^ mice retinas in the period of PD20-PD30^51^. Complex I deficiency triggers OXPHOS dysfunction and oxidative stress which cause cell death of the most sensitive cells, bipolar and amacrine cells, in the retina at an early age. This early cell death triggers the inflammatory response at PD30, which coincides with the loss of vision and will be followed by the loss of retinal ganglion cells at PD42^47, 52^. It has recently been shown that chronic hypoxia markedly improved survival, body weight, body temperature, behavior, neuropathology, and disease biomarkers in *Ndufs4* ^−/−^ mice^53, 54^. The systemic phenotype after selective inactivation of *Ndufs4* in neurons and glia cells (NesKO mice) is essentially identical to that of *Ndufs4* ^−/−^ mice, indicating the origin of this progressive neurodegenerative phenotype^47^.

In this paper, we report beneficial effects of the ROS-redox modulator small molecule KH176 in the ubiquitous OXPHOS complex I-deficient *Ndufs4* ^−/−^ mouse model based on the loss of brain microstructural coherence, increased lipid peroxidation as well as on the progressive neurodegenerative phenotype.

## Results

### KH176 maintains microstructural coherence in the brain of *Ndufs4*^−/−^ mice

To investigate brain microstructural organization in the *Ndufs4* ^−/−^ mice, diffusion tensor imaging (DTI) was performed with an ultra-high field MR system. Fractional anisotropy (FA) is often employed as a sensitive measure of microstructural coherence, being relatively high in white matter and lower in gray matter, which can be assigned to the presence of densely packed parallel oriented fiber tracts in white matter^55^.

**Figure 1:**
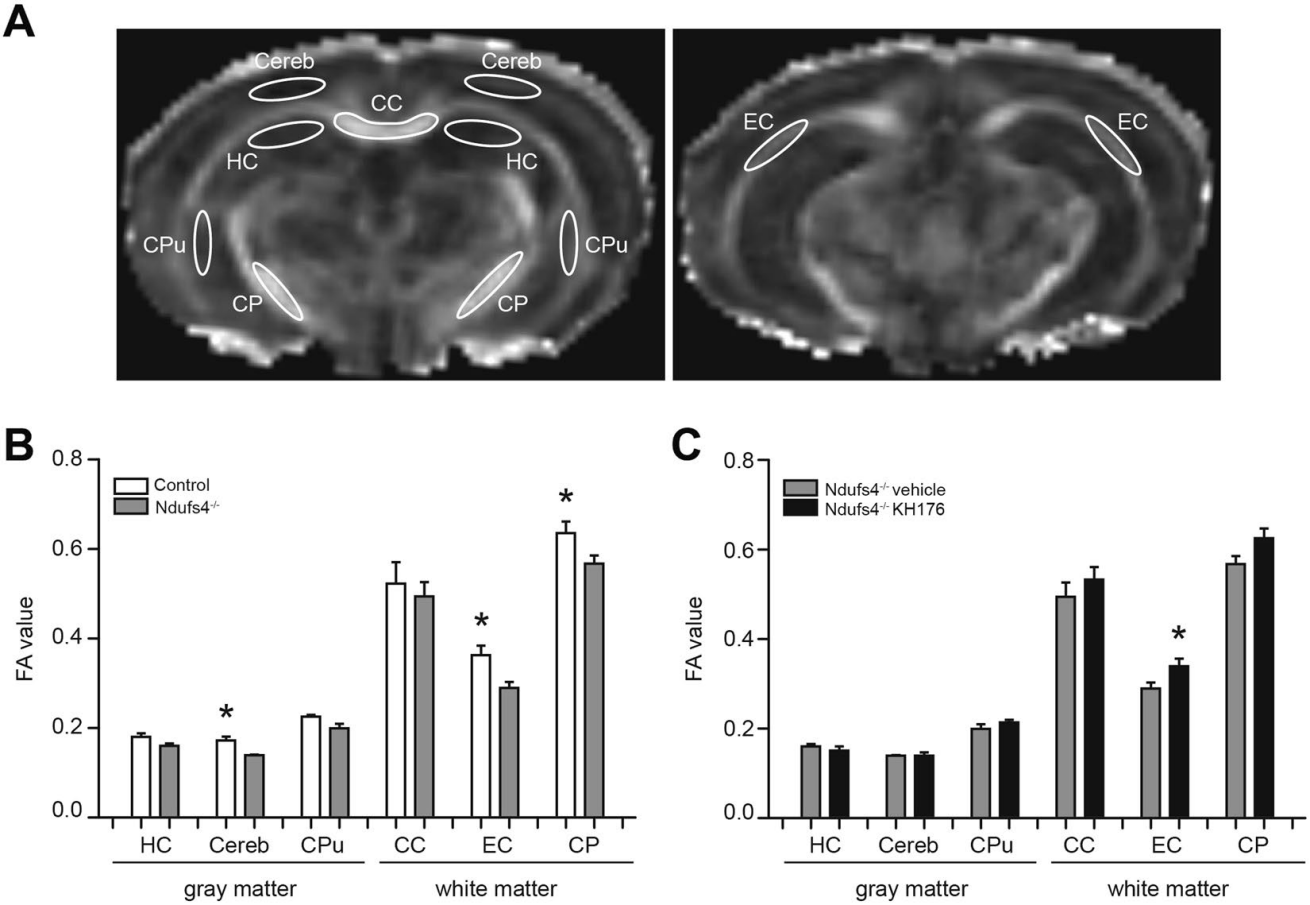
KH176 maintains microstructural coherence in the brain of *Ndufs4* ^−/−^ mice. (**A**) DTI was used to asses microstructural organization in the mouse brain. ROIs in gray and white matter are overlain on FA maps. CC, corpus callosum; Cereb, cerebral cortex; CP, cerebral peduncle, CPu, caudate putamen; HC, hippocampus; EC, external capsule. (**B**) FA values of control (white bar) and *Ndufs4* ^−/−^ vehicle-treated (gray bar) mice (n = 5–7) (PD > 42). (**C**) FA values of *Ndufs4* ^−/−^ vehicle (gray bar) and KH176-treated (black bar) mice (n = 6–7) (PD > 42). FA values expressed as mean ± SEM. Multivariate ANOVA (*p ≤ 0.05).

For calculation of the FA, six different brain regions of interest were defined (Fig. 1A). The FA values of the different white and gray matter brain regions in control mice were similar to those reported by others^55–57^. However, compared to controls, the FA values of *Ndufs4* ^−/−^ mice were significantly decreased in the cerebral cortex, external capsule and cerebral peduncle, indicating a loss of microstructural coherence in these areas (Fig. 1B). KH176 treatment in *Ndufs4* ^−/−^ mice resulted in statistically significantly higher FA values in the external capsule and a similar trend was found in the cerebral peduncle (Fig. 1C). This indicated that KH176 partially corrected FA values in *Ndufs4* ^−/−^ mice brain, and thereby maintained microstructural coherence in defined areas.

### Increased lipid peroxidation normalized by KH176

**Figure 2:**
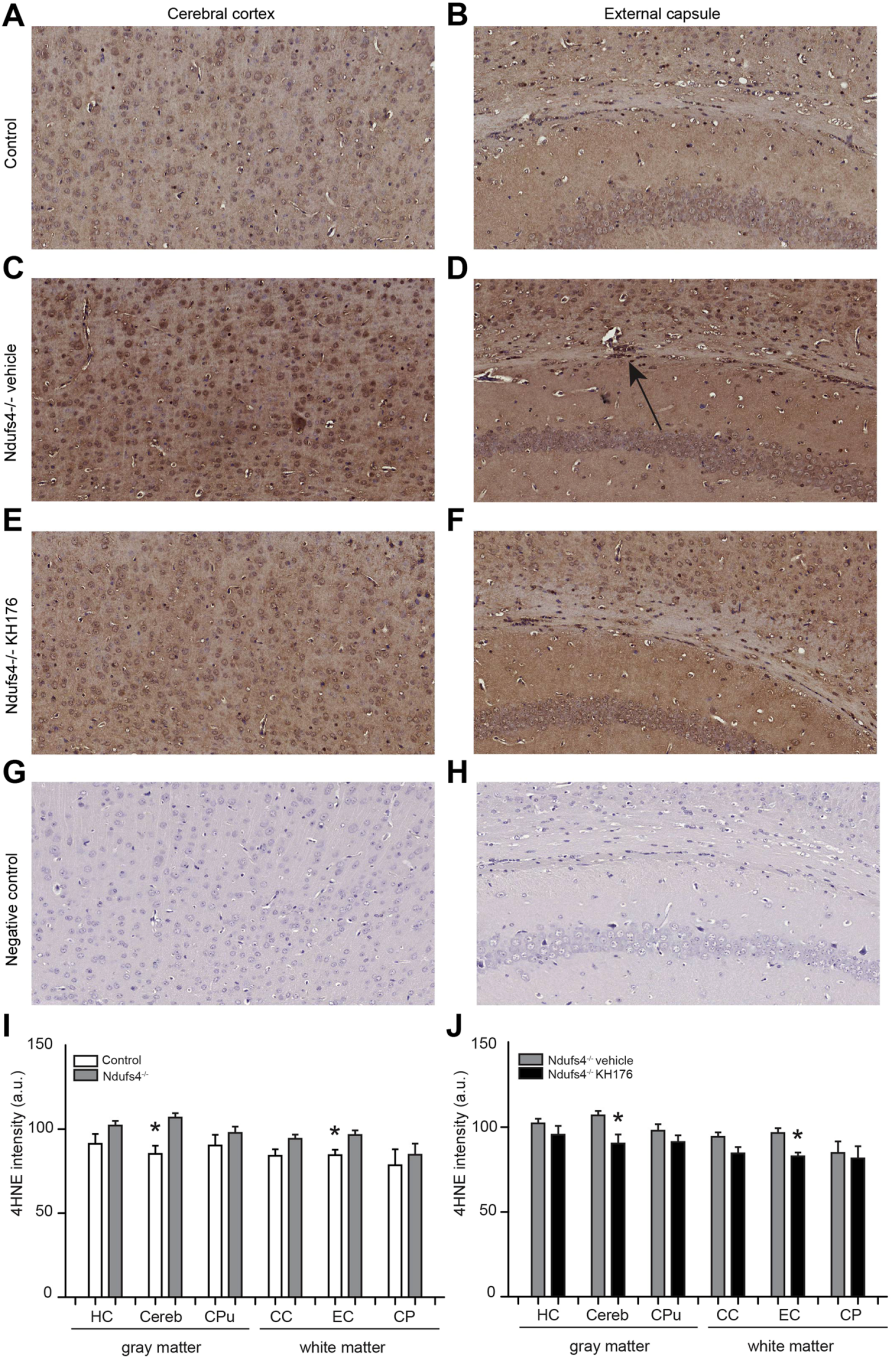
Increased lipid peroxidation is normalized by KH176. Representative images (20X) of 4HNE staining of the cerebral cortex (**A**,**C**,**E**,**G**) and external capsule (**B**,**D**,**F**,**H**) for a control, *Ndufs4* ^−/−^ vehicle-treated, *Ndufs4* ^−/−^ KH176-treated mouse and negative control. Arrow indicates the positive staining in the external capsule. (**I**,**J**) Quantification of the 4HNE staining of control (white bar), *Ndufs4* ^−/−^ mice vehicle (gray bar) and KH176-treated (black bar) (PD > 42). 4HNE (a.u.) expressed as mean ± SEM (n = 4). Multivariate ANOVA (**p < 0.01, *p < 0.05).

ROS are highly reactive molecules containing oxygen with unpaired electrons, which can lead to lipid peroxidation. The lipid peroxidation product 4-hydroxy-2-nonenal (4HNE) was used as an indicator for ROS, by immunohistochemical 4HNE staining as described previously by others^58^. 4HNE staining showed an overall increase in *Ndufs4* ^−/−^ mice brain compared to control (Fig. 2). This increase was statistically significant in the cerebral cortex and external capsule, which were the same brain areas with decreased FA values measured at PD > 42 (Fig. 2G). After KH176 treatment, lipid peroxidation was normalized (Fig. 2H). These results indicate a relation between FA and lipid peroxidation, which was confirmed previously in a study with bipolar patients^59^.

### Therapeutic effect of KH176 on brain histopathology in *Ndufs4*^−/−^ mice

An amino cupric silver stain allowed us to investigate the degree of neurodegeneration in the brain of *Ndufs4* ^−/−^ mice. A number of specific brain areas of both vehicle and KH176-treated *Ndufs4* ^−/−^ mice were positively stained (Supplementary Fig. S1). Spongiform lesions were evidently recognized within the interpeduncular nucleus and vestibular nuclei as centrally unstained areas (viii and xiii). Regarding incidence, distribution, morphologic characteristics and grade of the changes, largely comparable changes were observed in the vehicle and KH176-treated *Ndufs4* ^−/−^ mice. No histopathologic abnormalities were found in control mice. KH176 treatment was not able to reduce or prevent the severe brain pathology, including the vestibular nuclei lesions. Furthermore, KH176 treatment had no effect on disease onset or disease severity measured by phenotypic scoring or life span. Quintana *et al*. previously reported that inactivation of *Ndufs4* in the vestibular nuclei also induces breathing abnormalities and increased mortality, besides neurodegeneration^50^. Viral restoration of *Ndufs4* attenuated respiratory abnormalities and ameliorated the fatal phenotype of *Ndufs4* ^−/−^ mice. Vestibular pathology was still present in KH176-treated *Ndufs4* ^−/−^ mice, which explains the lack of extending life span in our study.

**Figure 3:**
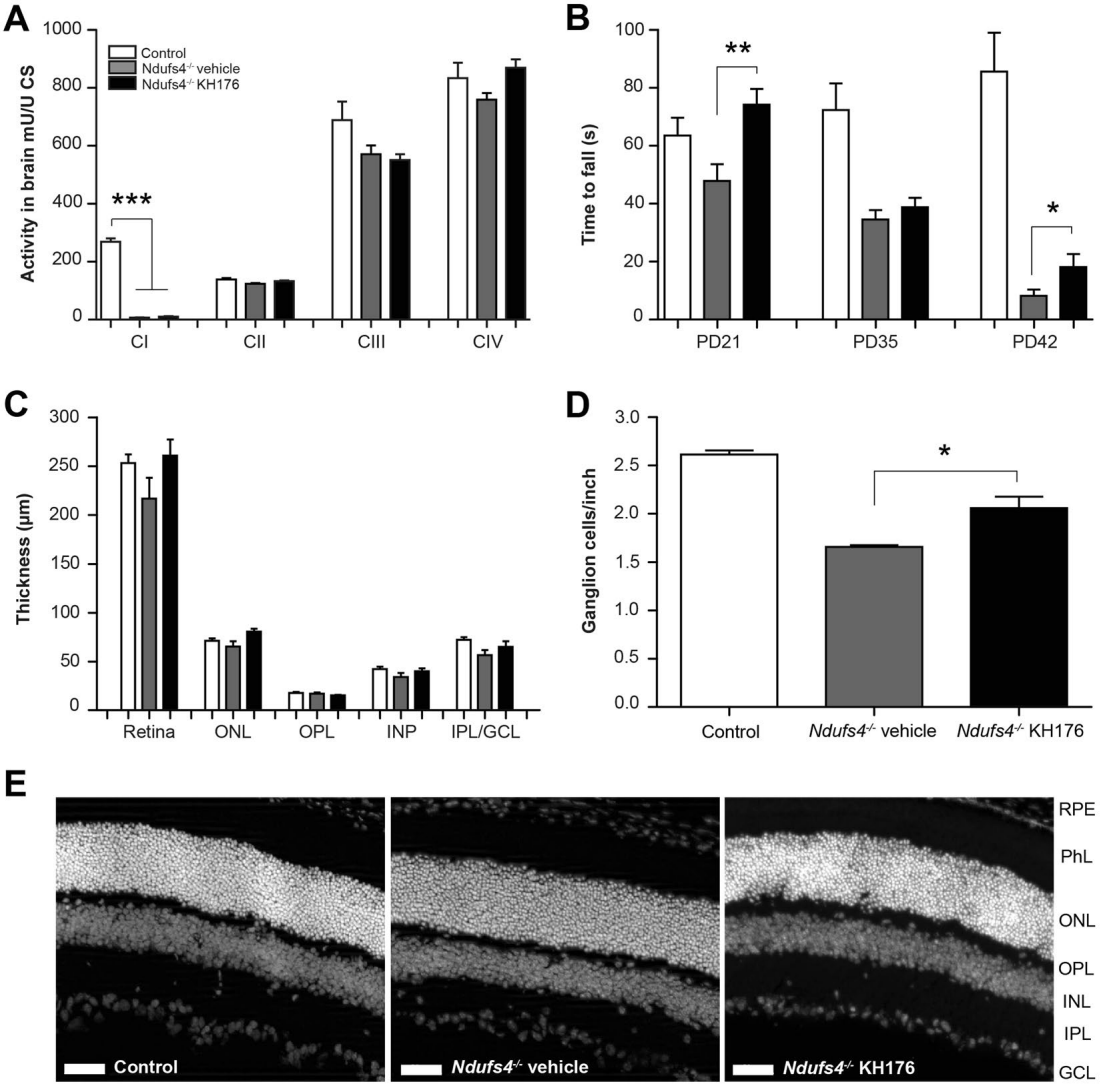
Improved motor function and reduced degeneration of ganglion cells in *Ndufs4* ^−/−^ mice. (**A**) Activities of the separate respiratory chain complexes were measured in whole brain homogenates of control (white bar), *Ndufs4* ^−/−^ vehicle-treated (gray bar) and KH176-treated (black bar) mice (at PD > 42). As expected, complex I was almost absent in brain tissue of *Ndufs4* ^−/−^ mice, however, detectable. Activity (mU/U CS) as mean ± SEM (n = 8). Multivariate ANOVA (***p < 0.001). (**B**) Rotarod performance of control (white bar), *Ndufs4* ^−/−^ vehicle-treated (gray bar) and KH176-treated (black bar) mice at PD21, PD35 and PD42. KH176 treatment significantly improved motor performance in *Ndufs4* ^−/−^ mice on PD21 and PD42. Time to fall (s) expressed as mean ± SEM (n = 15). Multivariate ANOVA (**p < 0.01, *p < 0.05). (**C**) Thickness of complete retina and its different layers; outer nuclear layer (ONL), outer plexiform layer (OPL), inner nuclear layer (INL) or the combined inner plexiform layer with the ganglion cell layer (IPL/GCL). Control (white bar), *Ndufs4* ^−/−^ vehicle-treated (gray bar) and KH176-treated (black bar) mice (at PD > 42). Thickness (µm) expressed as mean ± SEM (n = 3–4). (**D**) Number of retinal ganglion cells expressed as mean ± SEM (n = 3–4). Control (white bar), *Ndufs4* ^−/−^ vehicle-treated (gray bar) and KH176-treated (black bar) mice (at PD > 42). Kruskal-Wallis (*p < 0.05). (**E**) Representative images of the retina (scale: 50 µm). Different layers: retinal pigment epithelium (RPE), photoreceptor layer (PhL), outer nuclear layer (ONL), outer plexiform layer (OPL), inner nuclear layer (INL), inner plexiform layer (IPL) and ganglion cell layer (GCL).

The activities of the separate respiratory chain complexes were measured in whole brain homogenates of *Ndufs4* ^−/−^ mice (KH176 or vehicle treated) and control mice (PD > 42). As expected, complex I activity was almost absent in brain tissue of *Ndufs4* ^−/−^ mice, and complex III activity, like in patients, was slightly lowered. As the compound was specifically designed to target the two major cell biological consequences of complex I deficiencies, being elevated ROS and perturbation in redox signaling, as expected no treatment effects of KH176 were noted on OXPHOS complex enzyme activity (Fig. 3A)^38^.

### KH176 improves motor performance in *Ndufs4*^−/−^ mice

Rotarod performance, which measures motor coordination and motor learning, was significantly impaired in the *Ndufs4* ^−/−^ vehicle mice compared to control on PD35 and PD42 (Fig. 3B). Importantly, KH176 treatment in the *Ndufs4* ^−/−^ mice improved motor performance significantly on PD21 and PD42 (Fig. 3B).

In addition, the effect of KH176 on mitochondrial morphology and histochemistry of soleus muscle tissue was examined (Supplementary Fig. S2). Electron microscopy showed large mitochondria, some with abnormal cristae in both *Ndufs4* ^−/−^ mice groups. KH176 had no positive or negative effects in the soleus muscle, based on mitochondrial morphology and histochemistry.

### KH176 reduced degeneration of ganglion cells in *Ndufs4*^−/−^ mice

*Ndufs4* ^−/−^ mice present loss of vision at PD30, which coincides with increase of the retinal stress marker GFAP (glial fibrillary acidic protein)^52^. Long-term treatment (PD14–PD45) with 10 mg/kg/day resulted in retinal KH176 concentrations ranging from 486–523 ng/ml. No significant differences were noted in thickness of the different retina layers (Fig. 3C). Interestingly, the number of ganglion cells was statistically significantly reduced in *Ndufs4* ^−/−^ mice compared to control, and KH176 treatment statistically significantly reduced the degeneration of ganglion cells, although it was not completely halted (Fig. 3D and E).

### Therapeutic effect of KH176 on gait performance in *Ndufs4*^−/−^ mice

**Figure 4:**
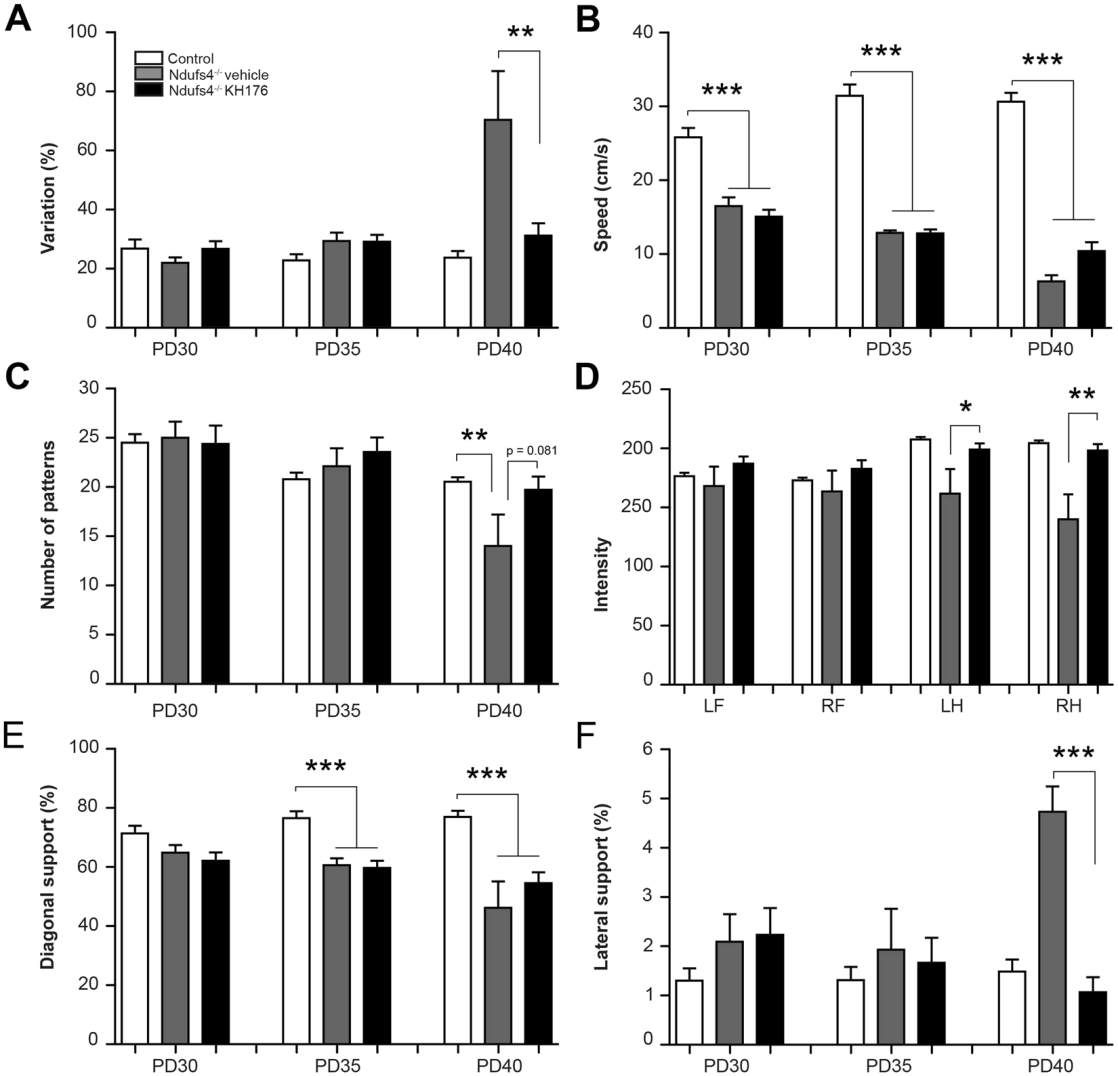
Therapeutic effect of KH176 on gait performance in *Ndufs4* ^−/−^ mice. (**A**) KH176 significantly reduced walking speed variability in *Ndufs4* ^−/−^ mice at PD40. (**B**) Run speed was significantly lower in *Ndufs4* ^−/−^ mice compared to control at all time points. (**C**) At PD40 the *Ndufs4* ^−/−^ mice showed a marked decrease in step sequence, indicating a more disorganized footfall pattern, which was prevented by KH176 treatment. (**D**) KH176 significantly increased maximum intensity of left and right hind paws at PD40. (**E**) Diagonal support was significantly decreased in both *Ndufs4* ^−/−^ groups compared to control at PD35 and PD40. (**F**) KH176 normalized lateral support in *Ndufs4* ^−/−^ mice at PD40. Control (white bar), *Ndufs4* ^−/−^ vehicle-treated (gray bar) and KH176-treated (black bar) mice (n = 11–15). Multivariate ANOVA (*p < 0.05, **p < 0.01, ***p < 0.001).

We evaluated the effects of KH176 intervention on gait abnormalities in *Ndufs4* ^−/−^ mice. Since walking speed differs during a whole run, the maximum variation of run speed was calculated (Fig. 4A). *Ndufs4* ^−/−^ mice are known to have increased walking speed variability at PD40, indicating a more hampered and uncoordinated gait^60^. KH176 treatment was able to prevent this, resulting in a more fluent and coordinated gait (Fig. 4A). Run speed was significantly decreased in *Ndufs4* ^−/−^ mice compared to control at all time points (Fig. 4B). KH176 treatment tended to increase run speed in *Ndufs4* ^−/−^ mice at PD40, however, not significantly. At PD40 the *Ndufs4* ^−/−^ mice showed a marked decrease in step sequence, indicating a more disorganized gait, which was prevented by KH176 treatment (Fig. 4C). Mean maximum intensity of the left and right hind paws on the glass plate was significantly lower in the *Ndufs4* ^−/−^ mice compared to control mice at PD40, which was not found in the KH176-treated mice (Fig. 4D). Relative duration of simultaneous contact with the glass plate was measured for different paw combinations. Diagonal support was significantly decreased in both *Ndufs4* ^−/−^ groups compared to control at PD35 and PD40 (Fig. 4E). In contrast, lateral support was statistically significantly increased in the *Ndufs4* ^−/−^ vehicle group compared to control at PD40, which was prevented by KH176 treatment (Fig. 4F). Altogether, these results show the ability of KH176 to improve abnormal gait in *Ndufs4* ^−/−^ mice.

## Discussion

In this study, we assessed for the first time DTI in *Ndufs4* ^−/−^ mice, a mammalian model for mitochondrial complex I deficiency. The observed reduced FA in the cerebral cortex, external capsule and cerebral peduncle in *Ndufs4* ^−/−^ brain, is in line with white matter diffusivity changes previously described in patients with mitochondrial disorders. Widespread reductions in FA (including the external capsule and cerebral peduncles) were found in three different clinical studies; in a group of pediatric patients, identified as having complex I or complex I/III deficits, among patients with m.3243A > G mutation and patients with *OPA1* autosomal dominant optic atrophy and Leber’s hereditary optic neuropathy^61–63^. Not only FA was affected in the cerebral cortex and external capsule, we also found a significant increase in lipid peroxidation in these brain areas. KH176 treatment was able to maintain microstructural coherence in the external capsule and to correct lipid peroxidation in both brain areas. Previously a relation has been noted between DTI measurements and peripheral measures of lipid peroxidation, suggesting that lipid peroxidation could be associated with the underlying pathophysiologic processes involving in white matter abnormalities^59^.

KH176 could not rescue the activity of isolated complex I. However, in isolated enzymatic assay the lack of activity of *Ndufs4* ^−/−^ complex 1 is mainly reflecting the lack of stability during the detergent-based isolation. In intact mitochondria of *Ndufs4* ^−/−^ mice the activity of complex I is only moderately affected. We could therefore not fully rule out a potential effect of KH176 on complex I activity which could explain its physiological beneficial effect.

Previous preclinical studies have evaluated the beneficial effects of other small molecule compounds in *Ndufs4* ^−/−^ mice. Treatment with the PARP inhibitor N-(6-oxo-5,6-dihydrophenanthridin-2-yl)-(N,N-dimethylamino) acetamide hydrochloride (PJ34) showed clinical effects based on neurological scoring, exploratory and motor activity, and rotarod performance, and similar to our study, no effect on life span was observed^26^. In contrast, rapamycin treatment resulted in a significant increase in life span, attenuated hindlimb clasping and maintained rotarod performance^25^. However, rapamycin dramatically reduced maximum body weight to 8–9 gram at PD35 (versus 12 gram at PD35 for vehicle *Ndufs4* ^−/−^ and 20 grams at PD40 for control mice) and no evidence for gain in quality of life was presented after PD50^25, 30^. KH176 was not able to prevent the development of the bilateral vestibular nuclei lesions, which contribute to lethality and explain the lack of extending life span up to the humane endpoint. However, the compound significantly reduced ganglion cell degeneration and improved different clinical disease symptoms, without any negative effects on developmental body weight. Furthermore, KH176 was able to improve rotarod performance, a measure of motor coordination and motor learning. *Ndufs4* ^−/−^ mice treated with KH176 showed a significantly more fluent and coordinated gait in contrast to the hampering and uncoordinated abnormal gait in vehicle treated *Ndufs4* ^−/−^ mice. We hypothesizes that the observed clinical effects of KH176 on rotarod and gait performance may be associated with the beneficial effects on ROS and brain microstructural coherence of white matter fiber tracts in the external capsule and cerebral peduncles. The external capsule consists of corticostriatal fibers moving to the basal ganglia, which plays a central role in reward, cognitive, and motor functions^64^. Furthermore, corticospinal and corticocerebellar fibers cross through the cerebral peduncles, involved in refining motor movements, motor learning and converts proprioceptive information into balance and posture maintenance.

In this study, KH176 was administrated twice daily via intraperitoneal injections resulting in two peak concentrations per day. For future studies the compound administration needs to be optimized aiming at an effective steady-state plasma concentration with less fluctuations. Since the *Ndufs4* ^−/−^ mouse is a severe disease model with limited time to rescue its fatal phenotype, it would be interesting to test the efficacy of KH176 in other species representing a less pronounced disease course.

In conclusion, these *in vivo* findings show that further development of KH176 as a potential treatment for mitochondrial disorders is worthwhile to pursue. Clinical trial studies to explore the potency, safety and efficacy of KH176 are ongoing (www.clinicaltrial.gov, NCT02909400).

## Material and Methods

### Animals

Initial breeding pairs of heterozygous *Ndufs4* ^+*/−*^ mice (mixed 129/Sv:C57BL6J background)^44^ were kindly provided by the Palmiter laboratory at the University of Washington and sustained in our breeding facility. *Ndufs4* ^+*/−*^ mice were intercrossed to produce *Ndufs4* ^−/−^ mice. Pups received toe tattoos for identification and tail clips were taken for genotyping at PD8. After genotyping *Ndufs4* ^+*/−*^ and *Ndufs4* ^−/−^ mice were randomly assigned to the KH176 or vehicle group and drug administration started at PD14. All mice were group housed under controlled conditions (temperature 20–22 °C and humidity 50–70%) with free access to standard food (Ssniff GmbH, Soest, 76. Germany. V1534-300 R/M-H) and water and maintained on a 12-h light/dark cycle. In our studies both female and male mice were used and equally divided. All mice were weaned at PD25. *Ndufs4* ^−/−^ mice were always housed with a minimum of one control littermate for warmth and provided with food on the bottom of the cage. Mice were weighed daily and clinical symptoms were observed. Based on ethical considerations and regulations it should be stated that no permission was obtained to pass the so-called humane endpoint, indicated by the severity of clinical symptoms observed in combination with a body weight loss of >20%. All animal experiments were in accordance with the Dutch laws (Wet op de Dierproeven), ARRIVE guidelines and European Community guidelines for animal care and approved by the Committee for Animal Care and Experimental Use of the University of Nijmegen.

### KH176

Out of hundreds newly engineered chemical entities, KH176 (or ((S)-6-hydroxy-2,5,7,8-tetramethyl-N-((R)-piperidin-3-yl)chroman-2-carboxamide hydrochloride; Patent WO2014011047 A1) was selected as the lead molecule based on an optimal balance between properties such as potency, stability, water solubility, oral bioavailability, blood-brain barrier permeability and metabolism. KH176 was dissolved in saline and administered with a maximal dose volume of 10 ml/kg bodyweight. Mice were twice daily i.p. injected, with KH176 (10 mg/kg/day) or vehicle, using 0.3 ml insulin syringes (30G × 8 mm needle) starting at PD14 until the end of the study. This dosing scheme was based on a pharmacokinetic and dose-selection study (Supplementary Fig. S3).

### *In vivo* imaging

MRI measurements were performed on an 11.7T BioSpec Avance III small animal MR system (Bruker BioSpin, Ettlingen, Germany) equipped with actively shielded gradients of 600 mT/m and operated by Paravision 5.1 software. We used a circular polarized volume resonator for signal transmission and an actively decoupled mouse brain quadrature surface coil for signal reception (Bruker BioSpin). The levels of anaesthesia and mouse physiological parameters were monitored following an established protocol^65^. Briefly, during the MR experiments, low-dose isoflurane was used (control mice 3.5% for induction and ~1.5% for maintenance, *Ndufs4* ^−/−^ mice 2.0% for induction and 0.5% for maintenance), slightly adjusted throughout the experiment to maintain a fast and stable breathing frequency (control and *Ndufs4* ^−/−^ mice >75 bpm). The mice were placed in a stereotactic device in order to immobilize the head. Body temperature was measured with a rectal thermometer and maintained at 37 °C for control and 35–36 °C for *Ndufs4* ^−/−^ mice by a heated air flow device. After standard adjustments and shimming, diffusion of water was imaged as described previously^55, 66^. In short, twenty axial slices covering the whole brain were acquired with a four-shot SE-EPI protocol. B0 shift compensation, navigator echoes and an automatic correction algorithm to limit the occurrence of ghosts and artifacts were implemented. Encoding b-factors of 0 s/mm2 (b0 images; 5×) and 1039 s/mm2 were used and diffusion-sensitizing gradients were applied along 30 non-collinear directions in three-dimensional space. Other imaging parameters: TR = 7.75 s; TE = 21.4 ms; field of view = 20 × 20 mm; image matrix = 128 × 128; spatial resolution = 156 × 156 × 500 µm; total acquisition time = 18 min. The calculation of the commonly used DT-MRI parameter FA, was performed following a protocol as described previously^55^. Briefly, the diffusion images were first realigned with SPM mouse toolbox, to compensate for small movement artifacts; thereafter, the datasets were spatially normalized to a study-specific template through linear affine and non-linear diffeomorphic transformation using ANTs. Following these pre-processing steps, the diffusion tensor was estimated for every voxel using the PATCH algorithm^67^. Region of interest (ROIs) were drawn by bilaterally (left and right hemisphere) for the hippocampus, cerebral cortex, caudate putamen, external capsule and cerebral peduncle. For the corpus callosum, only one ROI was drawn. The selection of ROIs was partly based on the ROIs previously described^56^ and the anatomical mouse brain atlas (Franklin & Paxinos). No differences in FA values were observed between the left and right hemisphere.

### Histology and electron microscopy

Brains were dissected and post fixed overnight in 4% PFA. Using MultiBrain® technology, twenty brains were embedded together into a single gelatin block and freeze-sectioned at 35 µm in the coronal plane through the area of interest. Areas of interest were based on the Franklin&Paxinos Mouse Brain Atlas. Amino Cupric Silver stain was performed, on every 12^th^ section spaced at 420 µm intervals, by the Neuro Science Associations services by standard methods. The 4HNE stain was performed in our lab by using the anti-4-Hydroxynonenal antibody (ab46545, dilution 1:100). Quantification of positive 4HNE staining was performed using Image J software.

Mouse eyes were enucleated, embedded in OCT (Optical Cutting Temperature compound, Tissue-Tek, Sakura Finetek, Torrance, CA) and frozen. Cryosections of 7 µm were dried for 1 h at room temperature, fixed in 2% PFA, washed in PBS, permeabilized for 20 min in 0.01% Tween in PBS and blocked for 30 min (0.1% ovalbumin and 0.5% fish gelatine in PBS). Anti-NeuN (Millipore, Billerica, MA) mouse monoclonal antibody was used as primary antibody (dilution 1:50 in blocking solution). Retinas were incubated overnight at 4 °C with primary antibody. Subsequently, sections were washed in PBS (3 × 10 minutes), incubated for 45 min at room temperature with the corresponding Alexa fluor-conjugated secondary antibodies (1:500) and DAPI (1:8000), washed in PBS (3 × 10 minutes), and mounted using Prolong Gold antifade kit (Life Technologies, Carlsbad, CA). Pictures were taken with Axio Imager (Zeiss, Oberkochen, Germany) microscope. Ganglion cell counting was performed using Image J software^68^ by measuring two parameters: i) the length of the layer by drawing a line following the ganglion cell layer (GCL) from one side of the picture until the other one and ii) the amount of DAPI and NeuN-positive cells in the GCL. Results were expressed in ganglion cells per inch ± SEM.

Soleus muscle for electron microscopy was fixed in 2% glutaraldehyde buffered with 0.1 M sodium cacodylate pH 7.4, post fixed in 1% osmium tetroxide in palade buffer pH 7.4 with 1% potassium ferrocyanide (K_4_Fe(CN)_6_.3H_2_0) and after dehydration in ethanol and propylene oxide, embedded in Epon. Semi-thin, 0.5 µm thick transverse sections were stained with 1% Toluidine blue. Ultra-thin sections were stained with uranyl acetate and lead citrate and examined in a JEOL 1400. Soleus muscle for histochemistry was snap frozen, PAS, Sudan Black, Gomori trichrome, NADH, SDH, COX and ATPase staining was performed following standard methods, described previously.

### Behavioral assessments

*Ndufs4* ^−/−^ mice (KH176 or vehicle treated) and control mice were scored on disease progression, rotarod performance, body weight, and life span (n = 12–15). Phenotypic scoring started at PD30 and consisted of three measurements, which were performed daily for the quantification of disease severity, including the Ledge test (direct measure of coordination), hind limb clasping (marker for disease progression), and kyphosis (characteristic dorsal curvature of the spine, caused by loss of muscle tone). Each measure was recorded on a scale of 0–3, with 0 representing an absence of disease phenotype and 3 representing severely affected. Three scores are combined in a total of 0–9 indicated as phenotypic score. The rotarod paradigm was designed to give a quantitative readout of motor capabilities in rodents. The rotarod (Hugo Sachs Elektronik/Harvard Instruments, Germany) accelerated from 4 to 40 RPM over the course of 5 minutes. The test consisted of a training period, during which the animals were exposed to the rotating rod, set at a constant 4 RPM. After the training period, the animals were tested a total of three times on the accelerating rotarod, with a 2 minute resting period in between each trial. The average time to fall from the rod (sec) was used as the readout parameter. Gait analysis was performed by using the CatWalk (Noldus, Wageningen, The Netherlands), which is an automated system to objectively assess neurological function in rodents. At PD21, mice were habituated to the CatWalk with their cage mates for 10 min during four consecutive days. Weekly testing was performed individually in the dark, starting at PD30. A session was successful after completing four compliant runs, which met the criteria set at a maximum run duration of 15 seconds. Run speed and maximum variation of run speed were calculated. Six different normal step sequence patterns can be recognized in rodents, depending on the sequential placement of the four paws. Step sequence represents the number of patterns that fall within the normal step sequence patterns and is used as an indicator of inter-limb coordination. Mean maximum intensity for each paw was calculated at PD40. Relative duration of simultaneous contact with the glass plate was measured for different paw combinations.

### LC-MSMS

The LC-MSMS system consisted of an HPLC (Accela®, Thermo Scientific, San Jose, CA, USA), a quaternary ultra high pressure pump, a vacuum degasser and an autosampler, coupled to a (TSQ Vantage®, Thermo Scientific, San Jose, CA, USA) triple quadrupole mass spectrometer. Chromatographic conditions: Liquid Chromatographic (LC) separation was performed using a Zorbax Eclipse Plus C18 analytical column (Rapid Resolution HD 1.8  μm; 50 × 2.1 mm, Agilent, USA) coupled with a UHPLC Guard Zorbax Eclipse Plus C18 pre-column (1.8 µm; 5 × 2.1 mm, Agilent). The mobile phase consisted of solvent A (0.1% (v/v) formic acid (HCOOH) in water) and solvent B (0.1% (v/v) formic acid (HCOOH) in acetonitrile). Chromatography was performed at an oven temperature of 40 °C. The sample injection volume was 10 µL and the analysis run time was 5 minutes. The samples were stored at a tray temperature of 15 °C. Mass Spectrometric Conditions: the compound-dependent parameters were optimized for the target compound to achieve the highest instrument response. MS parameters using positive ion mode were optimized to achieve good sensitivity for the compound in one single analytical run. The operating conditions were optimized by direct infusion of a 1 µM mixture of all analytics. Heated electrospray ionization (HESI) was operated at a spray voltage of + 3.0 kV, a capillary temperature of 200 °C and a vaporizer temperature of 390 °C. Nitrogen was used as sheath and auxiliary gas with a gas pressure of 40 and 35 AU (Arbitrary Units), respectively. Argon was used as collision gas at a pressure of 1.5 mTorr. During the LC–HESI–MS/MS analysis, a time-segment program was developed to switch the divert valve of the mobile phase to waste or detection mode to prevent ion suppression and contamination of the ion source. The analytics were monitored in selected reaction monitoring (SRM) mode.

### Respiratory complex assays

The activities of the individual respiratory chain complexes, citrate synthase (CS) and total protein in the brain tissue homogenates were measured spectrophotometrically, as described before^69, 70^. Measurements were only accepted if each of the duplicate values was within a 10% range of their average.

### Statistical analysis

All data were expressed as mean ± standard error of the mean. Kruskal-Wallis or multivariate ANOVA tests followed by bonferroni post-hoc testing for multiple comparisons were used (*p < 0.05, **p < 0.01, ***p < 0.001).

### Data Availability

The datasets generated during and/or analyzed during the current study are available from the corresponding author on reasonable request.

## Electronic supplementary material

Supplemental information
