# Supplementary material for: Therapeutic effects of the mitochondrial ROS-redox modulator KH176 in a mammalian model of Leigh Disease
Source: Sci Rep. 2017 Sep 15;7:11733. doi: 10.1038/s41598-017-09417-5 (PMC5601915; doi:10.1038/s41598-017-09417-5)
Supplement: Supplementary file 1 — Supplemental information [file 41598_2017_9417_MOESM1_ESM.pdf]

Supplementary information

Title: Therapeutic effects of the mitochondrial ROS-redox modulator KH176 in a mammalian model of Leigh Disease

Authors: Ria de Haas, Devashish Das, Alejandro Garanto, Herma G. Renkema, Rick Greupink, Petra van den Broek, Jeanne Pertijs, Rob W.J. Collin, Peter Willems, Julien Beyrath, Arend Heerschap, Frans G. Russel, Jan A. Smeitink

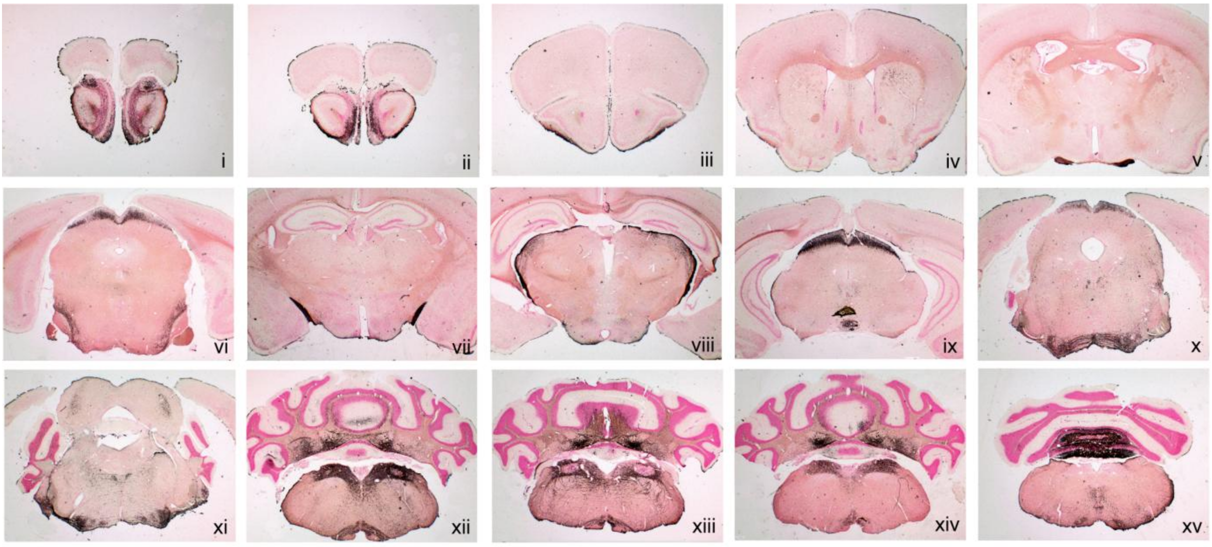

Figure S1: (A) Overview of positive Amino Cupric Silver (dark/black) staining (PD>42), which was recognized in a number of specific brain areas of both *Ndufs4*$^{-/-}$ groups. Affected areas included: i,ii) Olfactory bulbs; iii, iv) no findings (some false positive staining at the edges); v, vi, vii) Optic chiasm, optic tract; viii) Superior colliculus, Interpeduncular nucleus;, ix) Superior colliculus, Lateral Lemniscus; x)Superior colliculus, Trapezoid body; xi) Ventral spinocerebellar tract ;xii) Vestibular nuclei, Inferior olivary complex; xiii) Vestibular nuclei, Cochlear nuclei, Cerebral nuclei, Inferior olivary complex ; xiv) Vestibular nuclei, Fastigial nuceli ; xv) Nodulus (X) and Uvula (IX) granular and molecular layer.

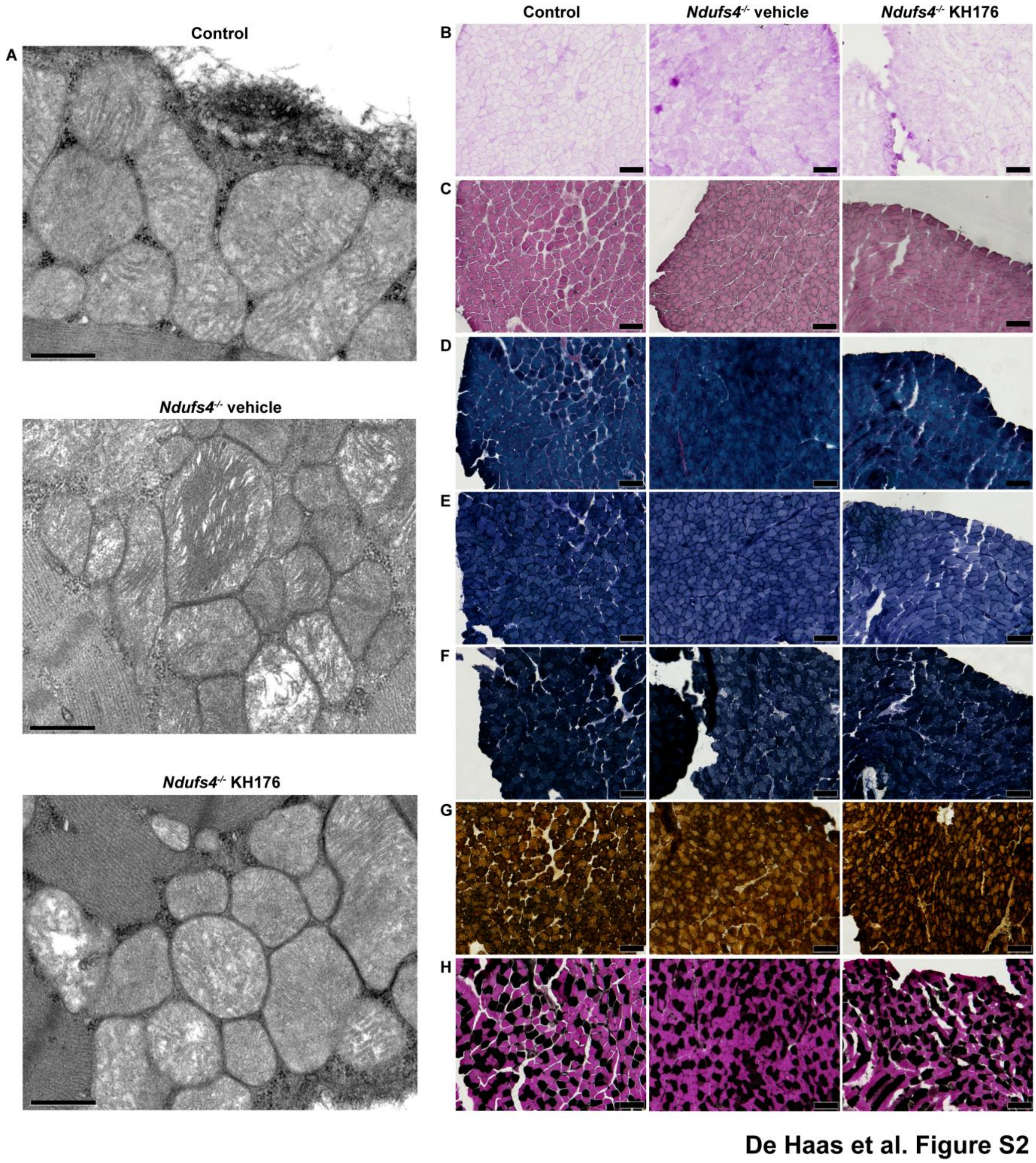

Figure S2: Mitochondrial morphology and histochemistry of soleus muscle.

(A) Electron microscopy images of soleus muscle (scale: 0.5 µm). *Ndufs4*$^{-/-}$ mice showed large mitochondria, some with abnormal cristae. No marked differences in morphology were observed between *Ndufs4*$^{-/-}$ vehicle or KH176-treated (PD>42). (B-H) Light microscopy of soleus muscle (scale: 100 µm). Histochemistry showed no abnormalities. (B) periodic acid-schiff (PAS) staining, (C) Sudan Black staining, (D) Gomori trichrome, (E) NADH, (F) Succinate dehydrogenase (SDH), (G) cytochrome oxidase COX and (H) ATPase staining. KH176 did not show any positive nor negative effect in the soleus muscle based on mitochondrial morphology and histochemistry (n=4).

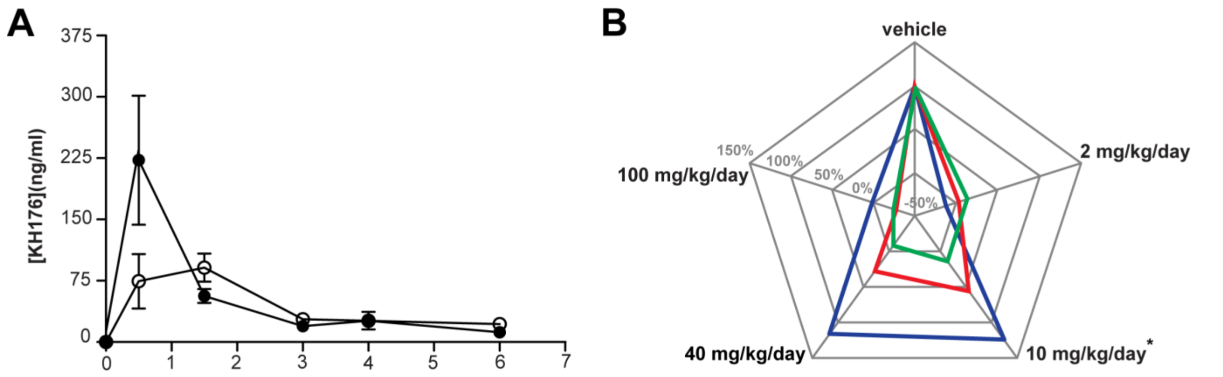

De Haas et al. Figure S3

Figure S3: KH176: Pharmacokinetic- and dose selection study

(A) Pharmacokinetic study of KH176 (single 5 mg/kg intraperitoneal dose) in *Ndufs4*$^{+/-}$ mice (n=3 per time point) showed maximum plasma concentration of 220 ± 140 ng/ml at 30 minutes (black circle) and brain concentration of 90 ± 30 ng/ml at 1.5 hours (white circle). (B) Dose selection based on relative rotarod performance (latency to fall) in *Ndufs4*$^{-/-}$ mice (n=7 per group), in a twice daily intraperitoneal dosing regimen based on its pharmacokinetic properties. Four different concentrations of KH176 were used (2mg, 10mg, 40mg and 100mg/kg/day), the *Ndufs4*$^{-/-}$ vehicle group was set at 100%. Behavioral testing was performed at PD21 (green line), PD35 (red line) and PD42 (blue line). *10mg/kg/dose was determined as the most clinical effective dose and used for all further experiments.
